# Editorial Notes

**Published:** 1905-09

**Authors:** 


### Editorial Notes.

---

**The British Medical Association, Leicester, 1905.**[1]

THIS Association has now recorded the seventy-third of its annual meetings, but it is the first visit it has paid to the historic and rapidly-growing town on the banks of the Soar, formerly known by the Britons as Caer Leir—the city by the Leir. It is by no means an unimportant part of the functions of this great Association that it gives its members annually an opportunity of learning something of the ancient and modern history and development of the principal cities of the empire. Leicester is now one of the most busy and thriving towns of the kingdom, with a population of nearly a quarter of a million, a commercial centre built up by many industries in addition to the well-known ones—boots and shoes, hosiery, and elastic webbing. The administration of the town by the Mayor and Corporation is one of the best illustrations of social co-operation, inasmuch as the gas, electric and water supplies, the arrangements for disposal of sewage, the isolation hospitals, the new cemetery with its crematorium, the numerous open-air spaces, including four large parks, four sets of public swimming baths, the municipal libraries, and the unsurpassed system of electric tramways, are all the work of this far-seeing and energetic body of civic rulers. Those members of the Association who were not engaged in the onerous duties of representatives had ample time, in addition to that spent at the sections, to see some of the many still existing evidences of the history of ancient times. Few places have a better record of the work of the Romans, for it is known that the Roman general, Ostorius Scapula, about fifty years after Christ, first established an encampment large enough for some 15,000 men on the rising ground above the river. This he named Ratæ Ceritanorum, the camp of the Ceritani, and the position

[1] For many of these details we have to acknowledge the assistance of Nuttall's *Guide*.

of the old Roman walls, afterwards existing for many centuries, is well known, although little trace of them now remains. In the year 120 the Emperor Hadrian is known to have passed through Leicester, a record of his tour having been found in the shape of a milestone with a laudatory inscription to this emperor upon it and the information "from Ratæ, two miles." After the Roman protection was withdrawn from Britain the Roman name Ratæ disappears, the primitive idea of a camp by the Leir was reinstated, and the chief city of the kingdom of Mercia then became known as Legro-ceaster, or Leicester, the camp on the Legre, or Leire. By this time the Angles had become Christianised, Mercia became a diocese, and the first bishop of Leicester was Cuthwin, who lived in 679, and whose church occupied the position of the present St. Margaret's. Doomsday Book gives a good account of what Leicester was at the time of the Norman invasion, and it was then that Leicester Castle was built by the governor of the town appointed by William the Conqueror. It was destroyed later and rebuilt by Robert de Beaumont, Earl of Leicester, in the time of Henry II., when the church of St. Mary de Castro and the Castle Hall, a great hall which still serves as the assize court of to-day, was first built. In the thirteenth century Sir Simon de Montfort, Earl of Leicester and Lord High Steward of England, who headed the barons in their struggle for freedom against the autocracy of the king, established the first Parliament of England in 1265. The great earl was killed at the battle of Evesham, but the Victoria Park of the present day remains as his gift. Not long after we find the title of Earl of Leicester passing to the younger son of King Edward III., John of Gaunt, who spent much of his time in Leicester Castle in the state befitting a prince of the blood-royal; for as the patron of literature and music and the friend of religious reform the Earldom of Leicester reached its zenith in one who was a son and a father of kings, a gallant soldier, a clever man, the friend of Chaucer and of Wycliffe. "At his death there were no more Earls of Leicester, for the earldom ceased . . . . because the glory of the earldom was merged in the glory of the throne."

The walks round ancient Leicester were conducted by Mr. S. Perkins Pick, F.R.I.B.A., who as an enthusiastic antiquary and a modern architectural authority was able to give the fullest information on both the old and the new. After taking the party through some of the modern buildings, he led them to St. Martin's Church, on the site of which once stood a temple dedicated to Diana, a Saxon church followed at the time of the Conquest, and the present church is Early English. The gravestones in the churchyard presented many interesting features in the evolution and decline of scroll engraving in the art of slate cutting and decoration.

The old town hall was pointed out to us as one of the most historic buildings of the town. It was originally the hall of the Corpus Christi Guild. At the west end of the hall is the mayor's seat, with the date 1586 and the initials of Queen Elizabeth, and in the mayor's parlour is the chair in which Prince Rupert sat when he had taken the town. All Saints' Church and St. Nicholas Church—Leicester's oldest building—standing on the site of a Roman temple dedicated to Janus, presented many points of interest. The stone pillars standing in the churchyard, unearthed during excavations in Holy Bones Street (a suggestive name), are clearly of Roman origin. Close by stands the well-known Jewry wall, probably part of the old temple of Janus, and two specimens of Roman pavement, both in their original positions and well preserved, are well worthy of a visit. These relics of the Roman occupation of the town are beautiful mosaic work of the simplest materials, one of them is said to be a floor in the residence of the Roman prefect, and about 1,800 years old.

A visit to St. Mary's Church followed. Here was formerly a Saxon church, built by Ethelflœda, daughter of Alfred the Great. This was destroyed by William the Conqueror in 1068, but was soon replaced by St. Mary de Castro, with which John of Gaunt was largely associated. The various styles of architecture at different periods are well seen—the original Norman added to by later Norman work, and then modified by the insertion of Early English arches. Leicester Castle of the present day shows little of its brilliant history in olden times, for here it was that the first meeting of King John's discontented barons was held in 1201, and from it the first Parliament of England was summoned by its earl, the great Simon de Montfort.

Any members passing De Montfort Square would have noticed the colossal marble statue of the late Rev. Robert Hall, M.A. (the work of John Birnie Philip in 1871), who removed from Leicester to Bristol in 1825 and died in 1831. The history of this distinguished orator—one of the greatest of his time—is of especial interest to medical visitors from Bristol. His grandson is with us as Chairman of the Committee of the Bristol Royal Infirmary, Mr. Robert Hall Warren.

Leaving the castle-yard by a picturesque turret gateway, we find ourselves in the district called "The Newarke," or "New Worke" of Henry, Earl of Lancaster, who built here his "New" or Trinity Hospital and his famous collegiate church. More modern skill has here constructed one of Leicester's most modern developments, the Technical and Art Schools, which rank among the first of the kingdom, and where all the sectional work of the meeting demands our attention.

As regards the meeting itself, the weekly journals have given full reports, and there is little need for comment. The presidential address of Mr. G. Cooper Franklin, F.R.C.S. Eng., endeavoured to improve the status of the profession by improving the details of medical education, a subject of perennial interest, often discussed, but mostly to little purpose. We trust that the excellent suggestions of the President may receive thoughtful consideration, and may be of benefit to future students of medicine.

The address given by Dr. Henry Maudsley, entitled "Medicine, Present and Prospective," affords material for much philosophic thought, for he foresees that medical science will play a large part in the future improvement of the race, and that the laws of mental breeding will do much towards that improvement.

The interesting and original address in surgery, given by Charles John Bond, F.R.C.S. Eng., was entitled "Ascending Currents in Mucous Canals and Gland Ducts, and their

Influence in Infection: a Study in Surgical Pathology." Mr. Bond's experiments were made on the large intestine, the female generative tract, the upper intestinal, the urinary, the respiratory tracts, and the ducts of glands. Many specimens in the museum gave the results on which his remarks were founded. It was an excellent piece of work well worthy of the occasion; its originality and importance cannot well be over-estimated, and the address is one of the things which will be remembered for years to come as emanating from the meeting at Leicester. The Pathological Museum gave much of Mr. Bond's careful work, for he, as chairman of the committee, had arranged that the museum should as far as possible illustrate the subjects under discussion in the various sections. His own contributions were very varied in character, illustrating not only his address, but extending over the whole realm of surgery. At the Oxford meeting it was thought and freely stated that such a standard of excellence in the Pathological Museum could not well be continued elsewhere, but Leicester has succeeded in maintaining a very high standard.

The sections, twelve in number, were the scenes of discussions and papers of the usual type. We propose to refer to only two of these—that of State Medicine and Laryngology. In the former of these Dr. Lionel Weatherly opened a discussion on the provision of sanatoria for poorer consumptives. He submitted that three classes of sanatoria were necessary, viz. institutions started and controlled by Boards of Guardians (only 18 per cent. of which were at present doing any substantial work in this direction), institutions for persons just above the pauper class, and, thirdly, for consumptives able to pay 25 to 40 shillings per week. In the discussion which followed, Dr. Shingleton Smith remarked that the public and the profession expected too much from sanatorium treatment, and that unless a careful selection of suitable cases could be made the method would soon fall into undeserved disrepute. Only pathologists could realise the hopelessness of expecting all cases of phthisis to be cured or even much improved by any kind of treatment.

In the laryngological section discussion on the treatment of laryngeal tuberculosis, introduced by Drs. Habershon, Jobson Horne and Barwell, the chief point of interest was the consideration of the earliest indications of the disease, hitherto regarded as predisposing causes, and the desirability of restricting operative treatment to a small and specially favourable class of cases, and of relying mainly on less radical measures. St. Clair Thomson and Watson Williams emphasised the importance and good results obtainable from sanatorium methods combined with absolute silence for a prolonged period.

The sections of Laryngology and State Medicine were associated in a discussion on diphtheria, with special reference to the infectivity and notification of the latent forms. Watson Williams drew attention to the relative frequency of very mild diphtheritic infection which, owing to the very slight symptoms, were prone to be overlooked unless special precautions were taken, these latent forms being nevertheless a frequent source of infection amongst contacts. He pointed out that the diphtheria bacillus is not only exceedingly polymorphic but variable in virulence, and that owing to the uncertainty as to the limits of normal variation of the diphtherial organism, it is difficult to define what must be regarded as infectious diphtheria. He rejected the view that the Hoffmann bacillus was an attenuated diphtheria bacillus. Special stress was laid on the frequency with which the nasal passages contained diphtheria-like bacilli, and in many cases this was not to be regarded as a source of danger; the importance of clinical manifestations as compared with bacteriological cultures could not be overlooked. Dr. Davies related the methods adopted at Bristol in determining infectivity, and considered that the presence of Hoffmann's bacillus in recent contacts necessitated special precautions and segregation.

The annual exhibition was held close by the sectional meetings in "The Newarke." It appeared to us to have fewer novelties than on previous occasions; surgical appliances appeared to predominate, and it was difficult to find amongst the drug and food exhibits anything which one had not seen before. The display of aseptic furniture, electrical apparatus, and motor-cars rather of the family coach type than for the country doctor on his rounds, occupied a great deal of the available space. It is satisfactory to find that the supply of special proprietary exhibits appears to be declining; it is impossible to use more than a small fraction of those on the market, and many of them are no better than the simpler preparations of the pharmacopeia. Regardless of the principles of scientific drugging, the prescriber appears to have drifted into the hands of the semi-scientific pharmacist in search of some proprietary compound to which a short but meaningless name may be attached, and it is too often the case that an elaborate but unscientific compound is selected by the prescriber instead of a more simple preparation of the drug he requires. The art of prescribing appears to be fast dying out; even physicians are adopting the ways of the surgeons by prescribing some well-known elaborate compound, the name of which is short and takes little time to write. The pharmacist makes his compounds elaborate in order that they may be the less easily imitated, but who can say what will be the combined effect of some twenty drugs in combination, even if the individual effect of each one may be foretold. Doubtless many of these combinations have proved themselves to be useful and have come into general use deservedly, but on the other hand there are many others whose only virtue depends upon an incessant and barefaced laudatory advertisement.

**Infant Life.**

In the seventy-five largest provincial towns of England the infant mortality rate during 1904 was 166; in Bristol it was 134. This means that out of upwards of 37,000 infants dying under the age of one year, Bristol contributed something over 1,200. The Infants' Health Society, in an interesting pamphlet on the "Present Conditions of Infant Life," points out the immense financial loss involved, not only in the high death rate, but in the physical and moral damage which present conditions inflict on the survivors, filling infirmaries, asylums, and prisons with maimed specimens of humanity, who have to be maintained at the public expense. This Society has established an Infants' Hospital in London, the first annual report of which has been published. From Dr. Vincent's medical report we gather that out of 183 cases treated 94 cases were discharged as having made good recoveries, or nearly 52 per cent. Seeing that the general condition of the 94 infants was but little better on an average than that of the remainder, this result seems very satisfactory. Dr. Vincent is a strong advocate of accurate percentage feeding, and his cases certainly give considerable support to the views he expresses. It is obvious, however, that hospitals cannot alone meet the needs of the situation, and the Infants' Health Society advocates various other measures, especially the establishment of milk depôts and dispensaries and the house-to-house visitation, which is generally associated with these institutions. In the pamphlet above mentioned the encouragement of breast feeding is very rightly put first as the most important of all measures for the protection of infant life, but it is recognised that under present conditions an enormous percentage of working-class mothers cannot feed their children naturally. To provide a suitable substitute for breast milk is, it is urged, financially impossible for many of these mothers, and hence the duty of providing such a substitute rests on the State, which is financially interested in the physical development of its infants collectively just as it is subsequently interested in their intellectual and moral education. In the last number of the *Journal* (vol. xxiii., p. 132) we printed an article dealing with milk depôts, in which the main outlines of a scheme were sketched whereby the value of these institutions might be more or less accurately gauged. The difficulty hitherto has been that no standard of comparison exists, and that the number of infants fed at even the largest depôts is too small to have any effect which can be statistically expressed on the infant mortality rate. It seems quite certain that the question of the infant death rate is becoming yearly of greater importance to us as a nation, and we commend a perusal of the publications of the Infants' Health Society to all who realise this fact and desire to assist in proper education of the public mind as to the best methods of dealing with the present calamitous state of affairs.

**Bristol Royal Infirmary.**

THE one hundred and sixty-ninth annual report, being for the year 1904, is much larger than on previous occasions, and is illustrated by many interesting photographs. It contains a pressing appeal from the President and Treasurer for additional annual subscriptions, and points out the stern necessity for raising a fund of £50,000 for renovations and improvements. The recent carnival, carried out with so much energy and skill by the President and his committee, has given an excellent start by clearing off the accumulated debt of twenty and more years. We trust that the appeal which Sir George White is about to make will meet with that response which his own enthusiasm and example deserve. He has already done much good work for this institution, and has shown that as President he intends to carry on that work with all the ability and zeal at his command.

